# Attending pediatric acutely ill patients at home: families’ socioeconomic characterization, expectations, and experiences

**DOI:** 10.1186/s12887-022-03724-1

**Published:** 2022-11-24

**Authors:** Astrid Batlle, Santiago Thió-Henestrosa, Imma Boada, Sandra López, Isabel Moya, José Carlos Fernández, Mariona Fernández de Sevilla, Juan José García-García

**Affiliations:** 1 Hospital Sant Joan de Déu, Barcelona, Spain; 2 Departament Informàtica, Matemàtica Aplicada i Estadística, University of Girona, Girona, Spain; 3 Graphics and Imaging Laboratory, University of Girona, Girona, Spain

**Keywords:** Acute pediatric homecare program, Families’ experience, Population characterization, Workload, Social network, Burnout

## Abstract

**Background:**

SJD a Casa is an acute pediatric hospital-at-home program that was initiated in 2019. For a thorough understanding of acute pediatric homecare programs, an analysis of all related factors, including the medical, social, and economic aspects as well as the family’s experience, is essential. However, no previous study has attempted a comprehensive evaluation of this topic in relation to a complex program such as ours (in terms of the diseases and treatments offered). In this study, we aimed to finely characterize the population that opts for pediatric homecare programs and obtain a thorough understanding of the families’ needs, which will improve our understanding of the program and potentially reveal possible deficiencies.

**Methods:**

This prospective quantitative and qualitative study involved collection of ordinal data as well as statements made by the caregivers of patients undergoing homecare. A total of 372/532 families were asked to answer two independent questionnaires (preadmission and postadmission) that evaluated their socioeconomic characteristics; expectations and experiences; and factors influencing the preference for homecare. The results were presented as frequencies and comparisons (Fisher’s exact test).

**Results:**

The families had an adequate social network and a less-than-expected workload, and most families responded that they would have repeated the experience despite the workload. The expectations regarding the caregiver’s well-being at home were better than the actual situation, since some caregivers experienced anxiety or fear. The rating for homecare was better than that for the inpatient care offered before the homecare transfer.

**Conclusions:**

Families included in the program were content with the homecare program and mostly responded that they would repeat the experience if needed. Although the duration of the program was short-term, some caregivers may experience symptoms of burnout like anxiety, which should be taken into consideration. Despite its limitations, this study offers the possibility of improving our service portfolio by focusing on vulnerable families’ access to the program and the caregiver’s risk of burnout.

**Supplementary Information:**

The online version contains supplementary material available at 10.1186/s12887-022-03724-1.

## What is known

- Pediatric homecare is an effective alternative to conventional hospitalization.^1^
- Pediatric homecare programs for acute patients are not frequent.^2^
- Families’ satisfaction with homecare programs has been described in previous studies.^3^

## What is new

- Families’ socioeconomic characteristics and caregivers’ experience of acute homecare is essential to gain a better understanding of an acute homecare program.
- The assessment of families’ expectations and actual experiences can facilitate the recruitment of new patients.
- Evaluation of the caregiver’s and child’s experiences can help the medical staff improve healthcare.

## Introduction

Home-hospitalization or hospital-at-home is described as home administration of medical care and treatment that is routinely provided in hospital settings. Home-hospitalization is a well-known, safe, and effective alternative to conventional hospitalization for eligible patients, as described in the literature [1–3]. Homecare, which is described as both health and social support received at the patient’s own residence, enables patients to be in their own environment while being accompanied and taken care of by their relatives. It offers significant benefits in comparison with inpatient hospitalization, including decreased iatrogenic complication and readmission rates and improved patient and caregiver satisfaction [4]. Experience in this field goes back to 1947, when the first adult home-hospitalization unit was designed in Guido Montefiore Hospital (New York). The main objectives of this program were to relieve hospital congestion and humanize hospital treatment [5]. After this experience, homecare has grown rapidly over the subsequent decades, mainly due to high healthcare costs and limited availability of beds in hospitals [6].

In pediatric practice, homecare experiences are primarily focused on patients with chronic diseases or those requiring palliative care [7–10]. Hospital Sant Joan de Déu (SJD) in Barcelona is one of the first hospitals that implemented, in 2019, a home-hospitalization program specifically addressing patients with acute illness and chronic exacerbations in Spain. The main objective of this program was to offer homecare for children with acute diseases, while maintaining quality and safety comparable to hospital care but in a more comfortable and pleasant habitat for patients and families.

The selection of appropriate families is as important as the identification of appropriate pathologies to be supported by the program. In this context, the ability to characterize the families that participate in acute hospitalization-at-home and evaluate their expectations and experiences is essential, since this information can help the hospital provide better care and potentially enroll more patients in the program. Although the characterization of families and their satisfaction with pediatric homecare has been well studied for patients with chronic diseases and those receiving palliative care [11–13], the corresponding data for acute pediatric hospital-at-home care are limited [14–18]. More specifically, no previous study provided extensive social information in relation to a complex acute pediatric home-hospitalization program like ours (considering the variety of pathologies and treatments offered).

One of the problems we face routinely is patient referral, since families may be frightened of getting involved in the care of the sick child and may reject hospital-at-home. Thus, when designing the study, two hypotheses arose: (1) the expectation is worse than the actual experience for the patient and caregiver; (2) families with limited resources may be more eager to adopt hospital-at-home care since it is easier to take care of siblings and other members of the family, and the extra costs of transport and food can be avoided. Nevertheless, aspects such as characterization of the families, expectations toward acute hospitalization-at-home, or the possible modulating factors when deciding to enter the program have not been reported yet. Moreover, a study of these factors can be challenging since the differences between pediatric chronic/palliative patients and acutely ill patients result in completely different environments: (1) chronic patients’ caregivers are well trained in the child’s care [19], while acutely ill patients are normally healthy children, who do not have such well-prepared caregivers; (2) acutely ill patients’ caregivers need to be skilled in nursing techniques in a short time; and (3) unlike acute patients, chronic patients have long-term relationships with their doctors, which may facilitate a successful medical process [20].

To address the limitations of previous studies on this topic, we conducted the present study that aimed to (1) describe the social characteristics of families participating in acute home-hospitalization; (2) assess families’ expectations toward acute hospital-at-home care; (3) investigate the factors that can influence the decision to prefer home-hospitalization modalities; and (4) evaluate families’ satisfaction with the program. The results of this study are expected to finely characterize the population that chooses acute home-hospitalization and provide a better understanding of the needs of such families, thereby improving our knowledge of this program and its possible deficiencies.

## Methods

### “SJD a Casa” program

SJD a Casa, which stands for SJD at home, is a homecare program for children who would otherwise be admitted in the hospital. This acute hospitalization-at-home program was designed in April 2019 at SJD in Barcelona, a tertiary hospital that receives relatively less complex cases from the adjoining regions and more complex cases from the rest of the country and overseas. The hospital conducts more than 25.000 discharges, approximately 238.000 outpatient visits, and 122.000 emergency visits per year. The aim of SJD a Casa is to ensure that patients receive the same care as that given in the hospital, but in a more comfortable environment, their own. Moreover, the hospital-at-home system can help free up beds for more complex and unstable cases at reduced costs.

Most of our patients specifically needed pediatric follow-up (not only nurse care). Diseases treated at home included respiratory illnesses requiring oxygen therapy; infections requiring parenteral antibiotic treatment; acute illnesses in patients with chronic diseases; and other pathologies that may demand pediatric follow-up, such as dehydrations, onset of nephrotic syndrome, or renal failure (requiring intravenous hydration).

This program involved two pediatricians and four nurses, in addition to administrative and technological support. The follow-up assessments included face-to-face visits and tele-homecare. The maximum capacity of the service was 12 patients. Most of the children were visited daily, while some required in-person visits two or three times a week. Attention was given 7 days a week from 8 am to 18 pm (pediatrician and nurse care during workdays, and nurse care on non-working days). Families had 24-hour telephone contact, and the patients were admitted to the Emergency Department if an evaluation was needed from 18 pm to 8 am.

Patients were referred to SJD a Casa from the Inpatient Ward, Outpatient Department, and Emergency Department, although most referrals were from the Inpatient Ward. The inclusion criteria were as follows: (1) age between 0 and 18 years with an acute or chronic exacerbated disease in need of hospital care, (2) living within 30 min of the hospital, (3) presence of a 24-h trained caregiver at home, (4) minimum habitability conditions at home; and (5) availability of telephone contact if necessary. Before being transferred to hospital-at-home, a specialized nurse from the home-hospitalization program met the patient and family to explain the main characteristics of the program, evaluate admission criteria, and instruct the caregiver to recognize warning signs and administer home treatments (intravenous antibiotics, oxygen therapy, continuous liquid infusion, wound care, etc.). Subsequently, the patient and family were transferred to home, where the family provided care since medical staff could afford to visit only once daily. Nurses oversaw the families’ training in taking care of the child and solved parents’ doubts concerning care at home. Pediatricians adjusted the treatments according to the patient’s evolution and determined the need for further explorations or the patient’s return to the hospital in case of deterioration.

### Participants

The study participants were the caregivers of patients who underwent the SJD a Casa program from 10/13/2020 to 12/15/2021. We included all patients admitted to the program from the Inpatient Ward. Participants were asked to answer two independent questionnaires voluntarily and anonymously. Patients admitted directly from the Emergency Department or Outpatient Department were excluded because they could not have answered some questions in the survey. The study was proposed to 532 families, of which 372 (69.92%) answered the first survey and 218 (40.98%) answered the second one. Since the questionnaires were answered anonymously and independently, we could not determine how many families answered both.

### Ethical considerations

Conflict of interest: none declared.

Informed consent was obtained from all individual participants included in the study. All procedures involving human participants were in accordance with the ethical standards of the Hospital Sant Joan de Déu research committee and with the 1964 Helsinki declaration and its later amendments or comparable ethical standards.

### Experimental design

In the first phase, an anonymous questionnaire in paper format was delivered in hand to the patient’s caregiver at the hospital bed before the patient began the hospital-at-home program. The caregivers answered the survey after the nurse provided them basic information on the program and care training. This questionnaire aimed to (1) evaluate the reasons why families choose acute home-hospitalization, (2) explore expectations regarding home-hospitalization (focusing on the child’s and the caregiver’s well-being); and (3) analyze possible factors that could influence the choice of homecare over in-hospital care (child’s anxiety and increased economic burden in the hospital). The preadmission survey has been provided in Additional file 1 (Online Resource 1).

In the second phase, another anonymous questionnaire was sent by e-mail (Google Survey) to the patient’s caregiver right after the hospital-at-home program. The survey was sent by e-mail to make it easier for the families to answer it. This survey aimed to (1) evaluate the families’ socioeconomic characteristics, (2) explore the current admission experience (child’s and caregiver’s well-being, economic burden, and convenience of hospital-at-home), (3) compare the attention received during home-hospitalization with that received during conventional hospitalization; and (4) analyze possible factors that could influence the choice of hospital-at-home care (previous admissions to hospital and traumatic experiences prior to current admission). The postadmission survey is provided in Additional file 1 (Online Resource 2).

Some of the questions were the same in the two surveys, since the aim was to evaluate whether the expectations and reality were consistent or whether the caregiver’s fears or beliefs may have distorted expectations. Since both questionnaires were voluntary and independent, some participants may have answered one or both of the surveys.

### Statistical analysis

Data were collected in an internal database. The results for each question were calculated in terms of absolute frequencies. Fisher’s exact test was used to compare preadmission results with postadmission results and home-hospitalization with inpatient hospitalization. For all analyses, we used the software R [21].

## Results

The principal findings are described below, while the results are more extensively described in Tables 1, 2, 3, 4, 5, 6, 7, 8 and 9. The results demonstrated that most of the families had an adequate social network. The workload was less than expected, and most families would have repeated the experience despite the workload. Expectations of the caregiver’s well-being at home were better than the actual situation, and some families experienced anxiety or fear. Families also rated homecare better than the inpatient care offered before the hospital-at-home transfer.

**Table 1:** Families’ socioeconomic characteristics

	Mean;sd or n;%
**Parent 1 age (*n*** **= 209)**	38.7;6.2
**Parent 2 age (*n*** **= 196)**	38.7;6.11
**Parent 1 origin (*n*** **= 217)**	
Africa	4;1.84%
America	36;16.59%
Asia	6;2.76%
Europe	11;5.07%
Spain	160;73.73%
**Parent 2 origin (*n*** **= 203)**	
Africa	4;1.97%
America	30;14.78%
Asia	5;2.46%
Europe	15;7.39%
Spain	149;73.40%
**Parents’ highest education (*n*** **= 214)**	
Elementary school	8;3.74%
High school	54;25.23%
College education	152;71.03%
**Familial economic characteristics**	
Receiving economic support (*n* = 218)	74;33.94%
Someone in family is unemployed (*n* = 214)	51;23.83%
Friends and family support if needed (*n* = 217)	153;70.51%
**Home characteristics**	
Patient has his/her own room (*n* = 215)	134;62.33%
Shared house with other families (*n* = 215)	3;1.40%
House well-equipped to accommodate hospitalization (*n* = 217)	213;98.16%
**Patients’ characteristics**	
Chronic condition (*n* = 215)	25;11.63%
Disability degree (*n* = 218)	10;4.59%
Level of dependence (*n* = 214)	11;5.14%
Work reduction (*n* = 213)	22;10.33%

**Table 2:** Reasons for choosing acute homecare and possible influencing factors

	n;%
**Reasons for choosing acute home-hospitalization (*n*** **= 372)**	
Child and family comfort	249;66.94%
Balancing work and family life	93;25%
Child’s health	75;20.16%
Economic burden caused by hospitalization	6;1.61%
Others^a^	18;4.84%
**Factors influencing the choice of acute homecare**	
Being admitted in a hospital previously (*n* = 217)	119;54.84%
Patient’s anxiety during examination in a hospital (*n* = 351)	131;37.32%
Increased economic burden during conventional hospitalization (*n* = 354)	243;68.64%
Previous traumatic event in a hospital^b^ (*n* = 217)	37;17.05%

^a^Other answers were “pediatric recommendation” and “because of COVID-19”

^b^Traumatic events included medical misdiagnosis, lack of empathy, and obstetric violence

**Table 3:** Expected and final experience during homecare

	**Expected experience** **(*n*** **= 372)** **n;%**	**Actual experience** **(*n*** **= 218)** **n;%**	**Fisher’s exact test** **(*p*** **value)**
**HOMECARE: CAREGIVER’S WELL-BEING**			
**Difficulties in contacting the healthcare team**			
Yes	12;3.26%	2;0.92%	0.094
No	356;96.74%	216;99.08%	
**Difficulties in administering medications**^**a**^			
Yes	2;0.55	14;6.45%	< 0.001
No	363;99.45%	203;93;55%	
**Workload**			
More^b^	57;16.19%	18;8.8%	0.091
Usual	236;67.05%	147;68.06%	
Less	59;16.76%	50; 23.15%	
**Feelings at home**			
Better	314;88.95%	171;79,17%	0.004
Equal	36;10.2%	40;18.52%	
Worse	3;0.85%	5;2.31%	
**HOMECARE: CHILD’S WELL-BEING**			
**Sleeping**			
Better	262;67.24%	169;77.52%	0.449
Equal	88;24.79%	48;22.02%	
Worse	5;1.41%	1;0.46%	
**Eating**			
Better	236;67.24%	163;75.02%	0.091
Equal	109;31.05%	53;24.42%	
Worse	6;1.71%	1;0.46%	
**Playing**			
Better	298;86.88%	197;91.63%	0.105
Equal	41;11.95%	18;8.37%	
Worse	4;1.17%	0;0%	
**Hygiene**			
Better	213;60.34%	160;73.73%	0.001
Equal	134;37.96%	57;26.27%	
Worse	6;1.7%	0;0%	

^a^In the preadmission questionnaire, when asked about problems during administration, parents reported the fear administering medications wrongly. In the postadmission questionnaire, 1/14 patients who had a problem when administering medication reported that it was not solved rapidly enough

^b^In the postadmission questionnaire, only 5/18 caregivers would not repeat the acute homecare experience because of workload

**Table 4:** Some of the answers to the open-ended question about the caregiver’s feelings at home (*n* = 40)

**Expressing fear/restlessness:**	
“I was afraid my boy could get worse, and I didn’t know what to do.”	
“I have felt a bit restless and nervous because of lack of confidence, as I knew I didn’t have a nurse 24 hours a day (…).”	
**Other feelings:**	
“I’ve felt happier because I’ve been accompanied by my family and the health team, which has helped professionally and humanly.”	
“I feel tired, but happy to see my child getting better.”	
“What really helped was not having to entertain a 1-year-old girl in a hospital bed. She seemed quieter in her own environment. The medication administration produced me some anxiety at the beginning, but having seen this in hospital, after a while everything went smoothly (...). I also was very grateful with the hospital’s training (...).”

**Table 5:** Caregiver’s opinion about the reasons why the child felt better at home and about recovery

**Homecare: Why the child felt better at home**	**n;%**
Because they were accompanied by their families and were in their own environment	177;84.69%
Because the child had improved when transferred to homecare	16;7.66%
Both	8;3.83%
Other answers^a^	8;3.83%
**Homecare recovery: Speed**	
The same or faster than that in the hospital	175;81.02%
Slower than that in the hospital	41;18.98%

^a^Other answers were as follows: “Because it is very difficult for her to eat (in hospital)”; “Because there’s no space in a hospital room to move, it’s really uncomfortable”; “She has everything at home: her bed, her bath, her hammock”; “He could rest at home, especially at night”

**Table 6:** Comparison of home-hospitalization and conventional hospitalization with respect to information and care

	Excellent n;%	Very good n;%	Good n;%	Regular n;%	Bad n;%	Very bad n;%	Fisher’s exact test (***p*** value)
**INFORMATION**							
**Home-hospitalization** **(*n*** **= 217)**	179;82.49%	32;14.75%	4;1.84%	2;0.92%	0;0%	0;0%	0.014
**Conventional hospitalization** **(*n*** **= 218)**	152;69.72%	49;22.48%	11;5.05%	5;2.29%	0;0%	1;0.46%	
**CARE**							
**Home-hospitalization** **(*n*** **= 216)**	184;85.19%	28;12.96%	2;0.93%	2;0.93%	0;0%	0;0%	0.004
**Conventional hospitalization** **(*n*** **= 216)**	157;72.69%	46;21.3%	10;4.63%	2;0.93%	1;0.46%	0;0%

**Table 7:** Comparison of home-hospitalization and conventional hospitalization in relation to the economic burden

	n;%
**Comparison of the economic burden in the hospital and in homecare (*n*** **= 205)**	
Higher in the hospital	124;60.49%
The same as that in the hospital	74;36.1%
Higher in homecare	7;3.41%
**Extra expenditure because of home-hospitalization (*n*** **= 218)**	
Yes	33;15.14%
No	185;84.86%

**Table 8:** Home-hospitalization convenience

	Mean;sd or n;%
**Rating SJD a Casa (*n*** **= 217)**	9.47;0.822
**Repeating homecare if needed (*n*** **= 215)**	
Yes	211;98.14%
No	4;1.86%

**Table 9:** Suggestions for improvement (*n* = 44)

**Related to the telemonitoring dispositive:**	
“Wireless monitoring dispositive.”	
“Being able to have the monitoring software in my phone, rather than having a tablet.”	
**Related to the healthcare team’s organization:**	
“To better specify visiting hours and medication scheduling, which must be difficult with so many children admitted to homecare.”	
“To include transportation to hospital when the patient needs to be checked there.”	
“Being myself mother and primary care physician, I think primary care should be contacted before discharge in case of chronic or complex patients.”	
“To answer the phone quickly.”	
**Related to medical devices used to administer medication:**	
“The nebulizer is too noisy.”	
“The nebulizer is too slow.”	

## Summary of reviewed articles

As mentioned previously, only a few studies have focused on understanding pediatric acute homecare and attempted to characterize the families choosing this form of care. The information described in these studies is summarized below.

Sartain et al. reported a qualitative study that aimed to compare 40 families’ experiences of homecare and hospital care. The project was based on a nursing acute hospital-at-home program, and the trial was limited to children with three types of symptoms: pyrexia (viral infection, tonsillitis), breathing difficulties (asthma, chest infection, croup), and diarrhea. The study obtained information on the parents’ and the patients’ opinions through interviews and studied user satisfaction, effects on the family, financial costs, and relationship with professionals. The primary results of their study indicated increased reassurance and confidence among parents in specific cases of acute nursing needs (no pediatric need in any case) [14].

Bryant et al. conducted a systematic review focused on inpatient versus outpatient parenteral antibiotic treatments at home, including assessments of efficacy, safety, satisfaction, and cost. The principal findings related to satisfaction were increased opportunity to keep up with school or work, greater privacy and comfort, improved quality of sleep and appetite, and increased time to spend with family [15].

Cabrera et al. evaluated a program similar to ours, which included pediatric and nurse care and covered a great variety of pathologies. Their study provided much information on the pathologies and treatments received at home, and limited information on perceived safety, satisfaction, and preference over conventional hospitalization. Nevertheless, it did not include the specific questions asked to caregivers, nor did it provide precise results. Greater comfort, privacy, ease of familial organization, and perception of earlier recovery were the main aspects to consider in this paper [16].

Young et al. reported a full qualitative study of children with subacute needs, based on interviews with 16 families. However, their program was based on tele-homecare (vital signs monitors, two-way videoconferencing connecting home and hospital, and community-based-homecare nurses–not hospital nurses or pediatricians). The principal findings suggested that care at home during the subacute care phase can be as good as that in the hospital, that families prefer to be at home, and that tele-homecare facilitated the transition home [17].

A previous study on our program, SJD a Casa, has also been reported, and that study aimed to conduct a pilot test evaluation to determine the program’s implementation in the hospital’s portfolio. The results were excellent (level of care scored “Excellent” overall, and all families expressed the desire to repeat the experience if needed) [18], as well as with previous work mentioned. However, the study used a restricted survey and a small sample.

Notably, none of these studies were like ours for the following reasons: (1) the studies did not aim to characterize the families of the patients; or (2) studies that reported a full description of the patient’s family had different program characteristics in terms of the pathologies treated and homecare staff.

## Discussion

### Actual work

Social characterization of families facilitates the identification of the predominant caregiver profile: Spanish parent, with college instruction, satisfactory household economy, and an adequate family and social network. Although these results may constitute a bias because of the experimental design (participants’ voluntariness to respond to the surveys), a point of interest is that the caregivers in our program were required to be involved in nursing techniques, some of them quite challenging, within a short time of empowerment. Thus, patients with fewer family resources may encounter difficulties entering the program.

An assessment of the reasons for choosing acute hospital-at-home care suggested that the economic burden of hospitalization was not a motivating factor in most of the cases. Nevertheless, more than a half of the participants agreed that the family’s economic burden in the hospital was higher than usual. Other expected modulating factors for selection of hospital-at-home (such as the child’s anxiety, a previous hospital admission, or the experience of a traumatic event in a hospital) were infrequent and do not seem to interfere with the homecare choice.

A focus on the caregiver’s experience may be valuable for such programs. Although the families confirmed that the workload was less than initially expected, the analyses of the caregiver’s well-being showed that the results were worse than the expectation at in the preadmission survey. This is remarkable, since it may suggest signs of fear, tiredness, and anxiety related to taking care of the child at home. Notably, although 18 families answered that the workload was more than expected, only five responded that they would not repeat homecare because of the increased workload. Thus, families’ desire for the patient to stay at home and the feelings of fear or anxiety among the caregiver could lead eventually to caregiver burnout.

Nevertheless, this study corroborated the previously reported satisfactory results for the acute hospital-at-home experience [14–16, 18]. The rating for hospital-at-home care was better than that for conventional hospitalization, and these results were consistent with previous studies [14, 15, 17]. This may be due to a closer relationship between caregivers and healthcare staff and because of the comfort of being at home.

The limitations of this study were as follows: (1) The participants’ willingness to respond the surveys and the different approaches for answering the surveys. While the first questionnaire was responded to in the hospital and followed by medical assistance at home, the second questionnaire was responded to when acute home-hospitalization was completed. Continuity in medical care may be related to more responses of the first survey. (2) The lack of socioeconomic information related to inpatient hospitalization precluded its comparison with the socioeconomic findings for hospital-at-home.

This paper offers a general vision of the socioeconomic situation of families admitted in an acute pediatric hospital-at-home program, and of the actual caregivers’ and child’s experiences. A future study with two dependent questionnaires may provide a better understanding of the feelings of each participant, instead of yielding only global data, and also provide information on patients who reject the program. Further studies of interest could focus on providing information on patients who reject the program, specific social aspects such as the experiences of single-parent families or immigrant parents, and the health staff’s view toward acute homecare.

## Conclusions

Despite the limitations of the study, this trial will help provide a thorough understanding of various aspects of an acute pediatric hospital-at-home system, which is essential to conduct an exemplary program. The findings offer the possibility of improving the service portfolio by highlighting the importance of focusing on vulnerable families’ access to the program and the caregiver’s well-being and risk of burnout. Our health staff has already started working on changes in the SJD a Casa program, with the main proposal being the elaboration of a brief survey to detect signs of caregiver burnout.

## Supplementary Information

**Additional file 1.**

## Data Availability

The datasets used and/or analyzed during the current study are available from the corresponding author on reasonable request.

## Abbreviation

SJD: Hospital Sant Joan de Déu

## Footnotes

1 Levine DM, Ouchi K, Blanchfield B, Saenz A, Burke K, Paz M, et al. Hospital-Level Care at Home for Acutely Ill Adults: A Randomized Controlled Trial. Ann Intern Med. 2020;172(2):77–85. 10.7326/M19-0600 González Ramallo VJ, Mirón Rubio M, Estrada Cuxart O, García Leoni ME. Usefulness of Hospital-at-home in nosocomial infections: advantages and limitations. Rev. Esp Quimioter. 2017;30:61–65 Leong MQ, Lim CW, Lai YF. Comparison of Hospital-at-Home models: a systematic review of reviews. BMJ Open. 2021;11(1):e043285. 10.1136/bmjopen-2020-043285

2 Pian J, Klig JE. Acute Pediatric Home Hospital Care. Pediatrics. 2020;145(6):e20190074. doi: 10.1542/peds.2019-0074. PMID: 32467093

3 De Zen L, Del Rizzo I, Ronfani L, Barbieri F, Rabusin M, Dall’Amico R, et al. Safety and family satisfaction of a home-delivered chemotherapy program for children with cancer. Ital J Pediatr. 2021;47(1):43. https://doi.org/10.1186/s13052-021-00993-x Castor C, Hallström IK, Landgren K, Hansson H. Accessibility, utilisation and acceptability of a county-based home care service for sick children in Sweden. Scand J Caring Sci. 2019;33(4):824–832. https://doi.org/10.1111/scs.12678
