# Supplementary material for: Attending pediatric acutely ill patients at home: families’ socioeconomic characterization, expectations, and experiences
Source: BMC Pediatr. 2022 Nov 24;22:679. doi: 10.1186/s12887-022-03724-1 (PMC9684951; doi:10.1186/s12887-022-03724-1)
Supplement: Supplementary file 1 — Additional file 1. [file 12887_2022_3724_MOESM1_ESM.pdf]

Attending pediatric acutely ill patients at home: Families' socioeconomic characterization, expectations, and experiences

BMC Pediatrics

Astrid Batlle[1]*, Santiago Thió-Henestrosa[2], Imma Boada[3], Sandra López[1], José Carlos Fernández[1], Isabel Moya[1], Mariona Fernández de Sevilla[1], Juan José García-García[1]

[1]Hospital Sant Joan de Déu, Barcelona, Spain

[2]Departament Informàtica, Matemàtica Aplicada i Estadística, University of Girona, Girona, Spain

[3] Graphics and Imaging Laboratory, University of Girona, Girona, Spain

***Corresponding author:** astrid.batlle@sjd.es

Online Resource 1. Preadmission survey.

PREADMISSION QUESTIONNAIRE

Reason for choosing acute hospital-at-home care

1. Choose the reasons why you decided to enroll your child to the hospital-at-home program (Multiple Choice):
    a. Because of my child's health
    b. Because of the economic burden that implies having my child admitted in hospital
    c. Because of the home comfort for my family and my child
    d. To improve familiar and labor conciliation
    e. If other, please write down why:

Expectations regarding the caregiver well-being at home

2. After the brief description you have received about how the program works, do you think you will have any problems to contact hospital-at-home professionals if needed? Yes/No
3. After the training in medication administration and/or the care needed at home, do you think you will have any problems administering medication?
    a. Yes. If this is your answer, please write down which problems are you thinking about:
    b. No
4. How do you think your workload will be during home-hospitalization?
    a. More workload than normally
    b. The same workload that I had prior to being admitted to hospital
    c. Less workload than normally
5. How do you think you will feel at home?
    a. Better than hospital
    b. The same as hospital
    c. Worse than hospital

Expectations regarding the child's well-being at home

6. How do you think your child will feel during hospital-at-home concerning:

	Better than hospital	The same as hospital	Worse than hospital
Sleeping			
Eating			
Playing			
Hygiene			

Possible influence factors

7. During medical visits in hospital, does your child feel anxiety, cries, does not let staff touch him or her? Yes/No
8. Do you think having your child admitted in hospital increases your economic burden? Yes/No

Online Resource 2. Postadmission survey.

POSTADMISSION QUESTIONNAIRE:

Social questions:

1. Parents' age
2. Parents' origin
3. Parents' educational background:
    a. Elementary education
    b. Secondary education
    c. University degree or more
4. Number of people living at home
5. Does the patient have his or her own room? Yes/No
6. Is the home shared with other people in addition to his family? Yes/No
7. Do you think your home is adequate to accommodate your child during hospitalization? Yes/No
8. How many people in your family are unemployed?
9. Do you receive any economic support?
    a. Social services support (dining scholarship, food aid, rental aid…)
    b. Government subsidy (unemployment, subsistence income, family subsidy)
    c. NGO (non-governmental organization)
    d. Other economic support:
10. Does your child suffer any chronic condition? Yes/No
11. Does your child have a degree of disability? Yes/No
12. Does your child have any level of dependance? Yes/No
13. Do you have a reduction of time worked because of a sick child (beyond current admission? Yes/No
14. Do you have family or friends to help with your child if needed? Yes/No

Current experience: Caregivers' well-being

15. Have you felt neglected during home-hospitalization? Yes/No
16. Have you had any problems administering medication? Yes/No
17. In case you had problems administering medication, has the problem been solved?
    a. Yes, rapidly
    b. Yes, but it took too much time
    c. No
18. How do you think the workload has been during home-hospitalization?
    a. The expected
    b. More than expected
    c. Less than expected
19. Knowing about the workload that implies home-hospitalization, would you repeat it if necessary? Yes/No
20. How have you felt during home-hospitalization?
    a. Better than in hospital
    b. The same as in hospital
    c. Worse than in hospital
21. You can specify how you have felt during home-hospitalization if you like:

Current experience: Child's well-being

22. How do you think your child has felt during hospital-at-home concerning:

	Better than hospital	The same as hospital	Worse than hospital
Sleeping			
Eating			
Playing			
Hygiene			

23. If you have answered "Better than hospital" in any of the cases, why do you think it is like that?
    a. Because he/she is in his/her own environment and accompanied by his/her family
    b. Because his/her health situation has improved
    c. Other
24. Do you think your child has recovered faster because of being at home?
    a. Yes
    b. No
    c. It has been the same

Current experience: Economic burden

25. Has it been an extra economic burden having your child admitted to home-hospitalization? Yes/No
26. How was the economic burden in home-hospitalization compared with conventional hospitalization?
    a. Indifferent
    b. Increased economic burden in hospital
    c. Increased economic burden at home

Current experience: Home-hospitalization convenience

27. Score from 1 to 10 the hospital-at-home program:
28. If your child needed to be admitted to hospital again, would you choose home-hospitalization? Yes/No
29. What do you think that can be improved in this home-hospitalization program?

Attention received compared with conventional hospitalization

30. Do you think your child would have been better cared of in hospital? Yes/No

31. If you answered "Yes" in the previous question, why do you think so?
    a. Because in hospital we have 24-hour on-site staff
    b. Because my child was not feeling well enough to be admitted to homecare
    c. Other
32. Please answer the next questions rating from 0=very bad to 5=excellent:
    a. How has it been home-hospitalization health staff attention?
    b. How has it been hospital health staff attention?
    c. How has information related to your child's health been explained to you during home hospitalization?
    d. How has information related to your child's health been explained to you during conventional hospitalization?

Possible influence factors

33. Has your child been admitted to hospital previously? Yes/No
34. Have you or anyone in your family come up against any bad experience in a hospital? Yes/No
35. If you have come up against any bad experience, please indicate which was.
